# Cognitive therapy for moral injury in post-traumatic stress disorder

**DOI:** 10.1017/S1754470X21000040

**Published:** 2021-01-13

**Authors:** Hannah Murray, Anke Ehlers

**Affiliations:** Department of Experimental Psychology, University of Oxford, Oxford, UK and Oxford Health NHS Foundation Trust, Oxford, UK

**Keywords:** betrayal, cognitive therapy, moral injury, PTSD, trauma

## Abstract

Moral injury is the profound psychological distress that can arise following participating in, or witnessing, events that transgress an individual’s morals and include harming, betraying, or failure to help others, or being subjected to such events, e.g. being betrayed by leaders. It has been primarily researched in the military, but it also found in other professionals such as healthcare workers coping with the COVID-19 pandemic and civilians following a wide range of traumas. In this article, we describe how to use cognitive therapy for post-traumatic stress disorder (CT-PTSD) to treat patients presenting with moral injury-related PTSD. We outline the key techniques involved in CT-PTSD and describe their application to treating patients with moral injury-related PTSD. A case study of a healthcare worker is presented to illustrate the treatment interventions.

**Key learning aims:**

(1) To recognise moral injury where it arises alongside PTSD.
(2) To understand how Ehlers and Clark’s cognitive model of PTSD can be applied to moral injury.
(3) To be able to apply cognitive therapy for PTSD to patients with moral injury-related PTSD.

## Introduction

Moral injury has been described as the profound psychological distress that can arise after perpetrating, failing to prevent, or witnessing events that transgress an individual’s moral or ethical code (Litz *et al*., 2009), including experiences of ‘betrayal of “what’s right”’ by leaders’ (Shay, 1994). Unlike post-traumatic stress disorder (PTSD), moral injury is not a mental disorder, but it can arise alongside, or contribute to the development of, PTSD as well as other mental health problems (Williamson *et al*., 2018).

The majority of moral injury research has been carried out with military veterans. Exposure to potentially morally injurious events is common during war, particularly responsibility for killing combatants and non-combatants and seeing, but being unable to help, injured civilians (Hoge *et al*., 2004). However, studies of other occupational groups have also revealed exposure to morally injurious experiences, including police officers (Komarovskaya *et al*., 2011), journalists (Backholm and Idås, 2015; Browne *et al*., 2012), child protection professionals (Haight *et al*., 2017), and medical students (Murray *et al*., 2018).

However, in our view, moral injury is not restricted to professional groups and these types of trauma. In our clinical practice, we have encountered moral injury reactions associated with various types of traumas. These have in common that actions or failure to act violated an important moral code of behaviour and harmed or betrayed others or failed to prevent harm. Both committing these actions or omissions and being subjected to them can lead to moral injury. Examples for actions include accidentally killing or injuring another person in a road traffic accident, offenders who hurt someone more seriously than they felt that person deserved (Evans *et al*., 2007), political prisoners who betrayed their friends under torture (Ehlers *et al*., 2000), soldiers who were involved in operations where women and children were killed, people who harmed others because of their political beliefs and later changed these beliefs, survivors of mass-casualty disasters, accidents or terrorist incidents who pushed or trampled other people in their panic to escape. Examples for failures to act to prevent harm to others include rape survivors who did not report a perpetrator who went on to rape others, doctors who missed a serious illness, people who did not help others who were calling for help during a natural disaster, and refugees who have fled a country where others are still being persecuted. Examples of being subjected to morally injurious behaviour of others include emergency workers who felt let down by their superiors during and after major incidents or people who were spied on by their friends or family.

The prevalence of moral injury outside the military has not been systematically investigated. There may be important differences between different populations which will depend on the individual’s appraisal of transgression of an important moral code within their social context. For example, part of the job as a soldier is to fight, including to harm and kill others. In order to do so, military personnel learn to overcome or suppress negative emotions associated with this, to potentially to dehumanise the enemy (Currier *et al*., 2015), and are supported by a system and culture that makes violence acceptable, and even honourable. However, there are different cultural norms and expectations in other occupational groups. For example, aid workers have the intention and desire to save lives, but may experience moral injury when they fail to do so, or when they have to make difficult ethical decisions about who deserves help where resources are being rationed. Indeed, different expectations and scenarios can arise within the same group; for example, some military personnel we have treated have experienced moral injury after killing an enemy combatant, but others experience no moral distress in this, but develop moral injury after another event, such as the death of a civilian or a fellow soldier that they failed to prevent.

At the time of writing, the COVID-19 pandemic has shone a spotlight on healthcare workers and carers who are facing ethical and moral challenges and potential moral injury (e.g. Williamson *et al*., 2020). Over-stretched health and social care services have led to challenges such as making decisions about the allocation of limited resources, staff working outside their usual roles, and staff being unable to contribute fully to their roles, such as when quarantined due to infection exposure or illness, or being hampered during emergency procedures by wearing personal protective equipment (PPE). A sense of betrayal by leaders who have inadequately prepared or supported staff facing the pandemic has been reported (Thomas and Quilter-Pinner, 2020). These staff are also facing the additional stresses caused by the pandemic, such as separation from friends and family, inability to engage in usual coping strategies due to the ‘lockdown’, fears for their own health and that of vulnerable family members and, possibly, bereavements.

At present, few treatment guidelines are available to assist psychological therapists treating PTSD where moral injury is an important part of the clinical picture. Addressing moral injury as part of prolonged exposure treatment for PTSD (Foa *et al*., 2007), and cognitive pocessing therapy (Resick and Schnicke, 1992), has been illustrated by Held *et al*. (2018). A brief CBT intervention for moral injury in veterans called adaptive disclosure (Steenkamp *et al*., 2011) has shown preliminary evidence for effectiveness in a small open trial (Gray *et al*., 2012).

Ehlers and Clark’s (2000) cognitive model of PTSD forms the basis of cognitive therapy for PTSD (CT-PTSD), a trauma-focused cognitive behavioural therapy recommended in international clinical guidelines (American Psychological Association, 2017; International Society of Traumatic Stress Studies, 2019; National Institute for Health and Care Excellence, 2018). CT-PTSD has demonstrated efficacy in randomized controlled trials (Ehlers *et al*., 2003; Ehlers *et al*., 2005; Ehlers *et al*., 2014) and in routine clinical practice (Ehlers *et al*., 2013). However, as yet, specific guidance on conceptualising moral injury using Ehlers and Clark’s (2000) cognitive model of PTSD, or applying CT-PTSD to people experiencing this difficulty, has not been published. In this paper, we will outline the conceptual and clinical issues in understanding and treating moral injury related to their PTSD using this cognitive model, and illustrate the treatment procedures with a case of a medical professional.

## A cognitive model of PTSD

Ehlers and Clark’s (2000) cognitive model of PTSD suggests that the core experience of PTSD is a sense of serious current threat even though the trauma is in the past. This perceived current threat can be external (‘I cannot trust anyone’; ‘The world is a dangerous place’; ‘People/organisations/the state will always let me down’) and/or internal (threat to sense of self, e.g. ‘I’m weak’; ‘I’m a bad person’; ‘If people treat me like this, I must deserve it’).

The sense of threat is maintained by three processes. The first relates to meanings that arise from the way an individual has appraised the traumatic event or its aftermath. For example, if patients now see themselves as incompetent, inferior or despicable, or other people as untrustworthy, this will create an ongoing sense of threat. If they felt betrayed by others or their employers, they may believe that they cannot trust anyone or that they wasted their life working for an organisation/person that betrayed them or behaved immorally.

The second concerns the nature of the trauma memory. The model suggests that when a trauma is processed in a predominantly sensory way (as a stream of sensory impressions) or as unreal/not happening to the self, the worst moments of the trauma are poorly elaborated and disjointed from other autobiographical information in memory. This accounts for the ‘here and now’ quality of PTSD memories; when they are recalled, people may be unable to access other information that could correct impressions or negative beliefs they had at the time, or make sense of their experiences. These types of memories are easily triggered by sensory cues similar to those encountered at the time of the trauma.

The third process maintaining the sense of current threat is the cognitive and behavioural coping strategies that the patient uses to attempt to reduce their sense of threat. These strategies can inadvertently increase symptoms (e.g. memory suppression or substance use) or the sense of threat (e.g. hypervigilance to danger). Importantly, avoidance, safety behaviours, social withdrawal, substance use and rumination prevent change (reappraisal) of traumatic meanings or in the nature of the trauma memory, which remains in its poorly elaborated state.

### Moral injury in Ehlers and Clark’s cognitive model

The experience of moral injury can be conceptualised using Ehlers and Clark’s (2000) cognitive model of PTSD. Exposure to potentially moral injurious events does not in itself cause PTSD; instead, it is excessively negative appraisals of the event in the context of an individual’s world view which leads to an ongoing sense of threat which may be internal (e.g. ‘I’ve let myself down’; ‘I’m disgusting’) or external (e.g. ‘other people will also betray me’; ‘if people know what I did, they will reject me’). Models of PTSD which have focused on fear-based trauma, and the role of habituation in its successful treatment, have been considered insufficient to explain the self-oriented negative moral emotions such as shame and guilt which are a central feature of moral injury (Litz *et al*., 2009). Ehlers and Clark’s (2000) cognitive model of PTSD explains a full range of emotional experiences related to idiosyncratic appraisals. For perpetration-based moral injury, excessively negative appraisals relating to over-estimation of personal responsibility and underappreciation of the role of the context of one’s actions (e.g. circumstances, own physical and psychological state, role of others), self-attack and expected contempt by others that lead to guilt and shame are especially relevant. For moral injury due to betrayal by others, appraisals related to unfairness, mistrust or permanent change that lead to anger and bitterness, and a sense of alienation from others (e.g. Ehlers *et al*., 2000) are central.

Some negative appraisals linked to moral injury may be entirely accurate, e.g. ‘I’ve taken someone’s life’ or ‘someone I trusted has betrayed me’. Ehlers and Clark’s (2000) model focuses on excessive appraisals that go beyond what everyone would find threatening. It is often the generalisation and extrapolation of meaning that represents an inability to accept and process morally injurious experiences within an individual’s self, world and others view which leads to an ongoing sense of threat, e.g. ‘I have lost my soul and no-one will ever forgive or love me’ or ‘I need to distance myself from others so that I cannot be hurt again’.

The model also includes the influence of previous beliefs and experiences in shaping appraisals. Patients affected by moral injury often report a strong ethical and moral code, which developed through earlier experiences, and cultural and systemic influences. For example, people in the military, emergency services and medical professionals often have extremely high personal standards, which make it harder to assimilate breaches to their moral and ethical standards. Previous experiences often include earlier traumatic events, which are common for people in occupations involving high exposure to trauma and potentially morally injurious events. Repeated exposure can build up layers of meaning, such as ‘there’s something wrong with me that I no longer feel upset by this’ or ‘this happening again proves that I am inferior’.

The nature of the trauma memory may also be important in understanding moral injury-related PTSD. Patients often report feeling numb and cut-off at the time of the trauma. If the trauma is related to their job, they are often in a ‘professional mode’, deliberately distancing themselves from emotions to cope with the difficult aspects of their work. Even outside of occupational-related experiences, people often describe dissociated, almost ‘out-of-body’ experiences during morally injurious experiences, which will affect the cognitive processing of the event. They may have been under maltreatment or pressure by others (such as during torture or prolonged abusive relationships) and have experienced mental defeat, the perceived complete loss of autonomy, which may be accompanied by the sense of not being human any longer (Ehlers *et al*., 2000) and is re-experienced when reminders of the event are present.

The resulting sense of threat will understandably lead to a range of cognitive and behavioural strategies intended to reduce the threat or the symptoms. People who have encountered moral injurious experiences at work often try to suppress their emotional reaction to be able to continue to function in their occupation. Other behaviours that have commonly been associated with moral injury such as withdrawal, rumination, substance abuse and self-harm, all prevent the reappraisal of negative cognitions and processing of the trauma memory.

**Figure 1.**
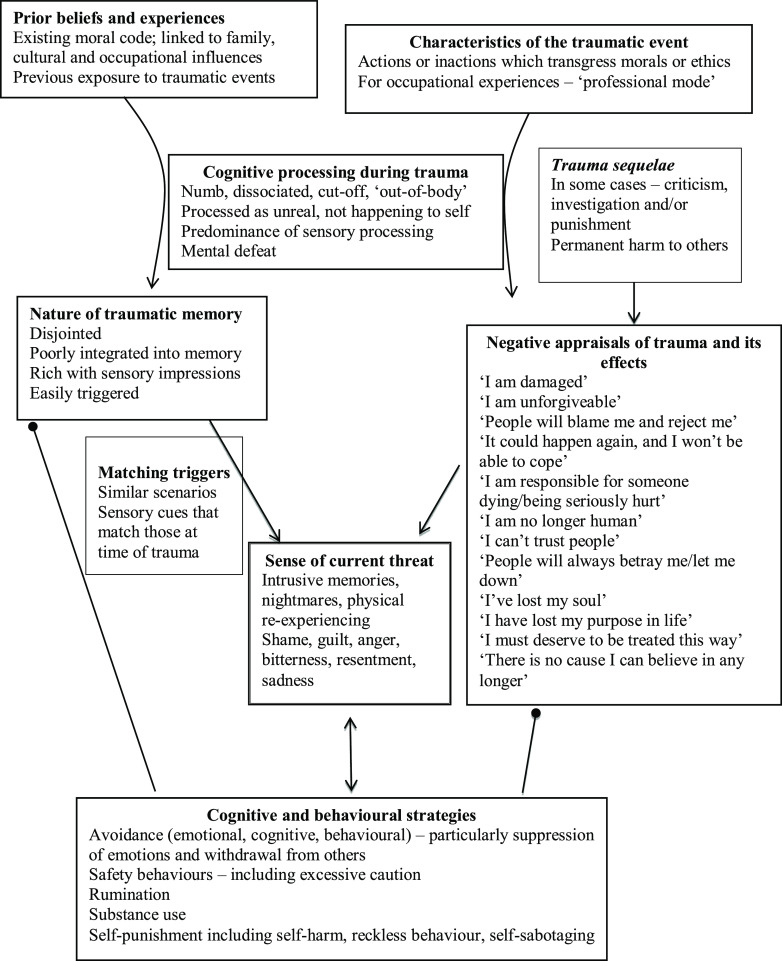
Ehlers and Clark’s (2000) cognitive model of PTSD, applied to moral injury (round arrowheads stand for ‘prevents change in’).

Figure 1 illustrates how typical features of moral injury-related PTSD are represented in Ehlers and Clark’s (2000) cognitive model of PTSD.

## Cognitive therapy for PTSD

Ehlers and Clark’s (2000) cognitive model of PTSD forms the basis of cognitive therapy for PTSD (CT-PTSD), a trauma-focused cognitive behavioural therapy. Treatment usually consists of up to 12 weekly sessions of up to 90 minutes, with up to three monthly follow-up sessions if patients reexperience a limited number of traumas. More sessions may be needed if multiple traumatic events are reexperienced.

In line with the model, the aims of CT-PTSD are as follows:

- To modify threatening appraisals (personal meanings) of the trauma and its sequelae.
- To reduce re-experiencing by elaboration of the trauma memories and by ‘breaking the link’ between everyday stimuli and trauma memories (‘then versus now’ trigger discrimination training).
- To reduce cognitive strategies and behaviours that maintain a sense of current threat.

For further information on how to conduct CT-PTSD, including training videos, questionnaires to guide treatment, guidelines for conducting treatment remotely and PTSD information leaflets, go to www.oxcadatresources.com. These training materials assume existing training and competence in CBT.

## Addressing moral injury in CT-PTSD

The core treatment strategies of CT-PTSD can all be used with patients with moral injury-related PTSD. The following suggestions are examples of how the core techniques can be applied with these patients. A summary table (Table 1) is provided. The order presented represents a typical course of therapy, but may be adjusted depending on the individual.

**Table 1.** CT-PTSD treatment strategies with moral injury applications

CT-PTSD treatment technique	Moral injury application
Psychoeducation and normalisation	Include psychoeducation on moral injury Normalise full range of peri-traumatic experiences Read others’ accounts of similar experiences and use these as part of Socratic dialogue
Individualised case formulation	Formulate role of peri-traumatic numbing and/or dissociation in inhibiting memory processing Discuss role of mental defeat in affecting view of self if applicable Explore appraisals and role of previous beliefs and experiences
Reclaiming your life	Incorporate reclaiming of values, self-identity and connections with others, self-care Address blocking beliefs, e.g. ‘I don’t deserve to be happy’
Updating the trauma memory	Generate updating information, e.g. context of traumatic situation (e.g. circumstances, own physical and psychological state, role of others) Introduce updates to trauma memory as soon as possible Initial work on important meanings leading to shame and guilt before accessing the trauma memory in detail if indicated, e.g. the patient is reluctant to discuss it or is at risk of drop-out
Working on meanings of the trauma and its aftermath	Identify and address distorted appraisals using guided discovery, responsibility pie charts, contextualisation, surveys, addressing thinking errors, psychoeducation, and seeking opinions of others Accept responsibility for genuine fault Consider costs and benefits of ongoing self-punishment and/or angry rumination Work on moving forward through making amends via apologies and restitution, including in imagery
Trigger discrimination	Review re-experiencing to identify triggers, including ‘affect without recollection’ Learn and practise ‘then versus now’ discrimination
Site visits	Consider earlier use if patients were dissociated at time of trauma or in a professional role Encourage patients to drop occupational role focus on visit Plan the visit ahead, particularly if it includes the patient’s workplace Use virtual site visits where returning is impractical
Address maintaining behaviours/cognitive strategies	Explore costs and benefits of strategies and experiment with dropping them Reduce substance use Prioritise self-punishing behaviours and revenge rumination if presenting a risk

### Psychoeducation and normalisation

In the early stages of CT-PTSD, we use psychoeducation to help patients understand PTSD and to normalise their symptoms and experience during the traumas. With moral injury, we also explain and normalise this experience, including the range of emotional reactions that people have during and after a morally injurious event. This is particularly important as patients with moral injury often withdraw and avoid talking about their experiences, so may have had little opportunity to challenge appraisals about their reactions (e.g. ‘I’m despicable because I lost control’; ‘I’m a monster because I felt numb’). We may provide psychoeducation about common reactions such as dissociation and disgust to illustrate that these are natural, automatic reactions to distressing events. Surveys can also be useful to normalise reactions and decrease feelings of shame or inferiority. This involves gathering a range of opinions, for example on how others view a person’s reactions.

Normalising information can also be found by reading first-person accounts of similar experiences. For example, several books have been written detailing personal experiences of moral injurious events, such as *What it is like to go to War* by Karl Marlantes, *A Soldier’s Song* by Ken Lukowiak, *Complications* by Atul Gawande (about making medical errors), *Journalists under Fire* by Anthony Feinstein and *999: My Life on the Frontline of the Ambulance Service* by Dan Farnworth. Various online resources can also be useful. For example, *Accidental Impacts* (accidentalimpacts.org) is a website for people who have caused serious accidents. If these resources trigger trauma memories for a patient, it is important to first work on ‘then versus now’ trigger discrimination (this technique is described later).

Such materials often help people feel less isolated. They can also be used as part of Socratic dialogue to access alternative perspectives, for example by asking the patient what judgements they make of the person they have read about, whether they deserve to be punished, and in what way, and what they would say to them if they had the opportunity. Patients often express a more compassionate view of others than they hold for themselves.

### Individualised case formulation

Another early task in CT-PTSD is developing an individualised case formulation with the patient. This is not as detailed as in Fig. 1 but includes a basic description of the main processes maintaining their PTSD (i.e. the sources of the sense of current threat and any problematic cognitive or behavioural strategies). With moral injury-related PTSD, we would include the role of dissociation or emotional suppression (whether deliberate or not) at the time of the trauma in inhibiting memory processing. Formulating also allows us to explore appraisals, link them back to previous experiences and beliefs, and to explain their role in perpetuating a sense of current threat. Inherent in this discussion is the message that appraisals are not facts, paving the way for cognitive restructuring work in later sessions.

### Reclaiming/rebuilding your life

Reclaiming previously valued and enjoyed activities or equivalents after a trauma is an important element of CT-PTSD, which starts in session 1 and is reviewed every session. Following moral injury, these assignments are also used to help patients reconnect with their values. The morally injurious event may greatly influence an individual’s sense of identity, so re-engaging with previously valued activities can draw attention to alternative meanings (e.g. ‘I am someone who cares about others’), promote the re-establishment of connections with others, and help people plan to live in accordance with their values in the future.

Working on ‘reclaiming your life’ assignments often reveals blocking beliefs such as ‘I don’t deserve to be happy’ and ‘I can’t trust other people’. These can be addressed in sessions with guided discovery techniques and behavioural experiments.

### Updating trauma memories

In CT-PTSD, important personal meanings (i.e. excessively negative appraisals in Ehlers and Clark’s model) are accessed by discussing the meanings of the worst moments of the traumas (‘hotspots’). These are identified by assessing the content of intrusive memories and through imaginal reliving or written narratives of the trauma memory. Updating information, which represents knowledge that was unavailable to the patient at the time of the trauma and puts the meaning of the hotspot into a less threatening perspective (e.g. ‘what I did was a mistake, it does not mean I am a morally defective person’) is then identified through discussion of the event and the evidence for and against the excessively negative personal meanings with the therapist, and integrated into the trauma memory as soon as it has been identified. Hotspots are updated by asking the patient to bring the hotspot to mind and then to remind themselves of the updating information, either during imaginal reliving or by reading through the hotspots in their trauma narrative that includes the updates (see training videos on oxcadatresources.com for more details). Early generation of updating information and linking it with the memory is especially important where moral injury has occurred, as self-attacking and highly aversive emotions such as shame may be triggered when the memory is accessed.

One advantage of recounting the trauma memory in detail is to understand more thoroughly the context surrounding the traumatic event. Also, a range of emotions and appraisals may have been experienced, including more straightforward appraisals during the trauma such as ‘I’m going to die’, which can be immediately updated. Appraisals related to moral injury, however, often require more detailed cognitive work (see next section). Where a patient is very ashamed about the event, experienced mental defeat, seems reluctant to disclose details of the traumatic incident, or is ambivalent about therapy and seems likely to drop out, we may work on the important cognitive themes associated with the experience before developing a detailed account of the memory. Once some helpful updating information (such as factors that explain their actions) has been identified, the trauma memory can be accessed in more detail with awareness of these updates.

As previously discussed, patients often describe feeling numb or dissociated at the time of a traumatic event, particularly if it occurs in the course of their work. It may be only later that distress arises, when the event is subsequently appraised. In some cases, events are re-appraised many years later due to a shift in circumstances or beliefs. For example, soldiers who experienced little distress at the time that they killed someone, as they were in a role where they felt justified and supported in the action, sometimes reappraise the experience many years later and develop delayed-onset moral injury and PTSD. In these examples, the updating of the peritraumatic appraisals may be less important than working on appraisals that developed since the event.

### Working with meanings

A key part of CT-PTSD is addressing the idiosyncratic personal meanings associated with the trauma. With moral injury, these often relate to the self or others being viewed negatively for their role in the event. One criticism of applying cognitive therapy to moral injury has been that it assumes that negative appraisals are distorted, whereas some judgements about transgressions in these cases may be accurate (Litz *et al*., 2009). However, as mentioned above, CT-PTSD aims to identify and change distorted appraisals; we would not seek to modify accurate ones. Where genuine responsibility lies with the patient, or another person, we work towards acceptance of this, and ways to move forward. However, often patients have over-estimated their (or another person’s) responsibility for an event, have discounted their physical or mental state at the time, or have generalised the meaning of it. Our first step, therefore, is to address these types of distorted appraisals using guided discovery. Strategies such as Socratic questioning aim to gently guide clients to explore and examine a wider range of evidence by asking questions that help them consider the traumatic situation from different perspectives, to generate less threatening alternative interpretations. The therapist works from a perspective of curiosity, rather than trying to undermine or prove the client’s perspective to be wrong. A non-threatening, collaborative style of working is essential. Some, or all, of the following specific techniques can be helpful.

#### Generating realistic appraisals of responsibility

Responsibility pie charts are useful for gaining a balanced perspective on individual responsibility. The patient is asked to list all of the people and factors that are responsible for the outcome, and allocate a ‘slice of the pie’ to reflect the size of their contribution. The patient is listed last. The goal is to provide a fair overview of responsibility, with the patient’s role, or the role of the person/organisation who betrayed the patient neither over- nor under-stated. For example, a soldier who reports 100% responsibility for killing a fellow combatant might be asked to list all of the other factors contributing to their death, such as the officer who gave the order, the other combatant (who presumably would have done the same), others who instigated the incident, the context of being at war, the people responsible for starting the war and so on. The soldier is allocated a slice of the pie for his or her actions but, given that they would not have killed the person for no reason, these other factors are taken into account when judging their responsibility.

#### Contextualisation

Another important area for intervention is where patients have failed to contextualise their, or another’s, actions in the situation they faced. Very often, potentially morally injurious events happen outside usual circumstances, so judgments of behaviour need to take this into account. For example, behaviour that would not be appropriate or acceptable in civilian life is necessary, encouraged and rewarded in a combat situation. A desire to intervene to help others may be denied, such as journalists who are instructed to observe and not intervene when reporting on a story, or overwhelmed by other needs, such as terrorist attack survivors who push others out of their way to escape, or medics who have limited resources to allocate in a crisis. Leaders who may not usually let people down may have faced unusual pressures or risks that influenced their behaviour. The therapist can help patients to understand the influences that context has on behaviour, such as the influence of cultural norms within groups (such as the military or gang membership), the impact of coercion or authority from people in powerful positions, the power of political ideology, the effects of physical deprivation, pain or discomfort (e.g. during torture) and the survival instinct, all of which can lead to people acting in ways they would not have predicted, or may feel are acceptable.

Socratic techniques can be used to help patients contextualise their and others’ actions or inactions, as can experiential exercises such as ‘zooming out’ of the memory in imagery, by imagining observing it from above. For example, a child soldier who had killed another boy as part of his induction into the rebel army was asked to view the incident as if an impartial observer, from above. He saw that he too had been a terrified child, whose life was being threatened when he took the action. As before, the aim of these exercises is not to convince the patient that they, or others, bore no responsibility for their actions if they did, but to view them in the context of an often extremely difficult (‘no-win’) situation.

#### Addressing thinking errors

Socratic techniques can also be used to identify and gently challenge thinking errors such as generalisation (e.g. ‘because I did this, it means I am rotten to the core’; ‘nobody can be trusted’) or superhuman standards (‘I should have been able to save them’, ‘I should have been able to predict what would happen’). Where generalisation has occurred, it is helpful to draw attention to the broad range of characteristics, experiences, actions and values that define a person. Following a betrayal of trust, it can help the patient to consider examples of times when they have not been let down. Continuum techniques (Padesky, 1994) can also be used to challenge black and white thinking, as people are rarely ‘100% bad’ or ‘100% good’ (see the training video on the OxCADAT resources website for a demonstration).

Litz *et al*. (2009) summarise the goal of cognitive work as generating ‘a new way to view the world and the self in it that takes into account the reality of the event and its significance without giving up too much of what was known to be good and just about the world and the self prior to the event’. In cognitive therapy, we achieve this by helping people to challenge excessively rigid views which make it hard to accommodate the morally injurious event. For example, if a patient expresses unrealistic standards, such as ‘I must never make a mistake’, a more flexible viewpoint can be developed which can incorporate the possibility that, as humans, we all make mistakes.

#### Psychoeducation on behaviour

Many people describe a sense of incomprehension about what people are capable of, whether atrocities they have witnessed or their own actions. Psychoeducation is often useful here, for example drawing on social psychology experiments like those of Milgram (1963) and Zimbardo (Haney, Banks and Zimbardo, 1973) to explain the capacity for humans to harm each other, given the right conditions (i.e. when instructed by people in authority and when placed in positions of power). These provide a useful alternative for the appraisal ‘because I have done this, it means I must be a psychopath/evil/a monster’ or ‘people are unpredictably violent’. It is also sometimes worth noting that someone truly ‘evil’ or psychopathic would be unlikely to experience remorse or emotional pain after an event. The BBC documentary *Five Steps to Tyranny* (2000, available online), describes the processes through which ordinary people end up committing acts of extreme brutality, and is a useful psychoeducational tool for patients who have witnessed genocide and war crimes-related atrocities.

Patients who are very self-critical about their peri-traumatic behaviour and responses may also benefit from psychoeducation to better understand them. For example, patients who experienced mental defeat can benefit from an explanation of learned helplessness and models of dissociation (e.g. Schauer and Elbert, 2010), to understand that ‘giving up’ can, in fact, be an appropriate and evolutionarily determined reaction to minimise harm in an inescapable situation.

#### Seeking the opinions of others

People with moral injury often withdraw, meaning they do not hear others’ opinions on what occurred. We can use surveys in some cases, although these are not always appropriate if events are outside the common realm of experience, or include very distressing details. Another useful technique is to ask the opinion of others in imagery. In ‘adaptive disclosure’, the patient selects a person whose opinion they respect, and who has ‘always had his/her back’ and has a conversation with them in imagery, explaining the situation and their feelings, and asking their opinion, such as how they can move forward (Litz *et al*., 2017). We have used similar imagery with a range of patients with different morally injurious experiences and have found it helpful, particularly with those who have judged themselves harshly, as they usually access a more compassionate viewpoint from someone they admire and trust. Following a betrayal, the imagery exercise gives the opportunity to vent distress and bitterness, and to receive an adaptive response, e.g. ‘you need to let this go – focusing on it is causing you pain’.

#### Acceptance and moving forward

For some patients, genuine responsibility and blame must be acknowledged. The conversation then shifts to how they can accept this and still move forward with their lives. Reviewing the costs and benefits of ongoing self-punishment often reveals that it benefits no-one. Instead, we discuss ways of moving forward by making meaningful change. For example, there may be opportunities to make amends for wrongdoing in some way (such as apologising, either in reality or in imagery, through a letter or conversation). Acts of reparation may also be appropriate when genuine harm has been done, such as taking action to benefit those directly affected, helping others in another way such as volunteering for a charity, or using imagery rescripting to repair in imagination.

Similarly, gestures and rituals can be a valuable exercise to commemorate an event or honour those affected, such as through funerals held in imagery, a symbolic act such as planting a tree or laying flowers or commemorating the anniversary of an event. Patients can also commit to living in accordance with their values in the future. For example, a driver who hit a child with his car returned to the scene of the trauma and laid flowers at the site. With the help of his therapist, he conducted an imagery exercise where he apologised to the child and their family and imagined the child ascending to heaven to be greeted by their grandparents. The driver committed to campaigning for safer driving practices, including reducing the speed limit in residential areas and near schools.

Moving forward from betrayal includes considering the pros and cons of holding on to anger, as ongoing rumination often increases the harm to the individual affected but not the person who betrayed them. We also consider ways to express anger and seek retribution. This may be in practical ways (such as making a complaint, reporting a perpetrator to the authorities) or by writing a letter (which may or may not be sent). Patients may also benefit from using imagery exercises including confronting the person or exacting some form of justice. However, any genuine plans for revenge should be risk assessed and discouraged.

### Trigger discrimination

Stimulus discrimination or ‘then versus now’ discrimination is used to address memory triggers in PTSD and to break the link between the trauma memories ‘then’ and the triggers to memories ‘now’. This includes careful review of re-experiencing episodes to identify triggers. Patients are often not aware that their trauma memories are triggered by sensory elements, such as colours, smells, tastes, sounds, postures and bodily sensations that match those present at the time of the trauma. For example, a sniper who shot a child approaching a military base had the memory triggered by slow steady breathing, which he used to control his shaking hands before making the shot. Triggers may also include media reporting of relevant topics and similar-looking places or people as those encountered during the trauma. Patients may simply be aware of a strong emotion arising seemingly out of the blue, without a trauma memory. This so-called ‘affect without recollection’ can initially be hard to spot, but as patients become more aware of their individual triggers, they gradually recognise these emotions as part of their trauma memories.

Once triggers have been identified, they are intentionally presented, memories and/or emotional reactions are elicited and the patient is encouraged to intentionally focus on what is different between ‘then’ (the trauma) and ‘now’ (the reminder). This ‘then versus now’ technique can be practised in session and for homework while deliberately introducing the trigger. Noises and pictures similar to those present at the time of the trauma can be found in internet sound libraries and Google Images. Bodily sensations can be recreated in session.

### Site visits

Returning to the scene of the trauma during treatment (ideally with the therapist) is used in CTPTSD to help elaborate the trauma memory, notice how the scene has changed and search for information that may help update appraisals. Where patients were dissociated or cut-off at the time of the trauma, this can be particularly important. It can also be an opportunity to make a symbolic gesture to mark the event. If a trauma was experienced while the patient was in a professional role, returning to the scene can also allow them to view what happened through a different lens. For example, a police officer returned to the scene of a suicide, where he believed he had behaved poorly by vomiting and delaying in attempting resuscitation. He was asked to come to the site visit in civilian clothes and to think how it would feel for any person to suddenly see a hanging body. He was able to see that his response was understandable and automatic, and reflected the fact that he was human, rather than meaning he was incompetent.

On a practical note, some site visits need to be arranged ahead of a session. If they include a person’s place of work, a more detailed discussion is needed about how to approach the visit with other members of staff. Sites that are impractical or unsafe to visit can be visited virtually using Google Street View, Google Earth and other internet resources. For more details on conducting site visits, see videos on the OxCADAT resources website, or read Murray *et al*. (2015).

### Address maintaining behaviours/cognitive strategies

Patients cope with the sense of current threat which is central to PTSD by adopting behavioural and cognitive strategies such as avoidance, hypervigilance and safety behaviours, which prevent the reappraisal of important beliefs, and the processing of the trauma memory. With moral injury, strategies often include emotional suppression and withdrawal from others, avoidance of similar situations, rumination and substance use. We work with patients to consider the costs and benefits of their strategies and experiment with dropping them. This often reveals further beliefs, for example, ‘If I don’t hold it together, I won’t be able to do my job’, ‘other people will think I’m a monster’ or ‘if I stop thinking about what happened, I am letting myself/them off the hook’, which can be addressed using cognitive restructuring and behavioural experiments.

Some people with moral injury and PTSD engage in self-punishment behaviours, including self-harm. Where these behaviours present a risk, they should be prioritised at the start of treatment. They are often linked to self-attacking beliefs such as ‘I deserve to be punished’ and emotions such as shame, which should be addressed as a priority. Rumination about revenge is common with betrayal-based traumas, and should also be prioritised, especially if there is a risk of harm to others.

### Issues for the therapist

Working with moral injury can be emotionally challenging for therapists. We may hear upsetting material that can challenge our own morals and ethics, yet a strong, non-judgmental therapeutic relationship is required to support our patients with disclosing their experiences. Therapists, therefore, need to reflect on their own reactions to moral injury cases and bring them to supervision. Prioritising self-care and team support are even more important than usual.

Therapists should inform themselves of guidelines around breaking confidentiality, in the event that patients report traumas that constitute a serious crime (such as murder, rape or child abuse). For example, the NHS code of practice states that confidentiality can be broken ‘in order to prevent and support detection, investigation and punishment of serious crime and/or to prevent abuse or serious harm to others’. However, decisions to break confidentiality should always be made on a case-by-case basis, weighing up the public good achieved by disclosure with the obligation of confidentiality towards the individual. Cases should be discussed with colleagues within the organisation. Guidelines vary in different countries; for example, in the USA, therapists have no obligation to report past crimes (only current or imminent harm to others), and are in fact prohibited from doing so by privacy laws.

### Limitations of the CT-PTSD approach to moral injury

The approach outlined in this paper builds on an evidence-based treatment for PTSD but has not been formally evaluated for moral injury and would benefit from rigorous testing and dismantling studies. The few studies that have been conducted with moral injury have focused on military samples, so extending these to a non-military population would be valuable.

The value of an individualised, formulation-driven approach like CT-PTSD is that it has great flexibility to adapt techniques to suit each client which, in our opinion, is appropriate for people presenting with moral injury given the breadth of experiences, consequences and reactions they face. However, this potentially makes it harder for therapists to learn and apply than a protocolised approach. This work should also be conducted under close supervision, not least to support the therapist in this emotionally challenging work.

Therapists will also commonly face obstacles in treatment. For example, individuals with moral injury often experience negative responses following their traumas from people within their social network and beyond. Unlike some traumas where there is a clear update to beliefs such as ‘it was not my fault’ or ‘the worst thing I feared did not occur’, the truth of some morally injurious events is more opaque and work on appraisals is more nuanced. Furthermore, some features of moral injury reactions can interfere with successful treatment, for example high levels of shame which make disclosure of traumas difficult, and which lead to beliefs such as ‘I do not deserve to feel better’. These will be challenges in all psychological approaches to moral injury, and require patience, persistence and compassion.

## A case study of moral injury – Tania

### Case description

Tania [name changed to maintain confidentiality] is a British Asian junior doctor in her thirties who developed PTSD following the death of a patient in her care. The patient had been a young woman, Queenie [name also changed], with a complex medical history who developed sepsis and went into cardiac arrest. Tania was responsible for her care at the time, but was also covering another ward during a busy night shift, and believed she had missed important signs that Queenie was deteriorating. Tania was experiencing severe symptoms of PTSD and depression at the time of assessment, and described a significant moral injury reaction, believing that she had failed Queenie and herself. She was also angry with the way the incident had been managed by the hospital. The on-call consultant had taken a long time to arrive, and she had been offered no support or opportunity to debrief after the incident. As well as perpetration-related moral injury, Tania had a strong sense of betrayal and felt that she could no longer trust senior staff to support her.

**Figure 2.**
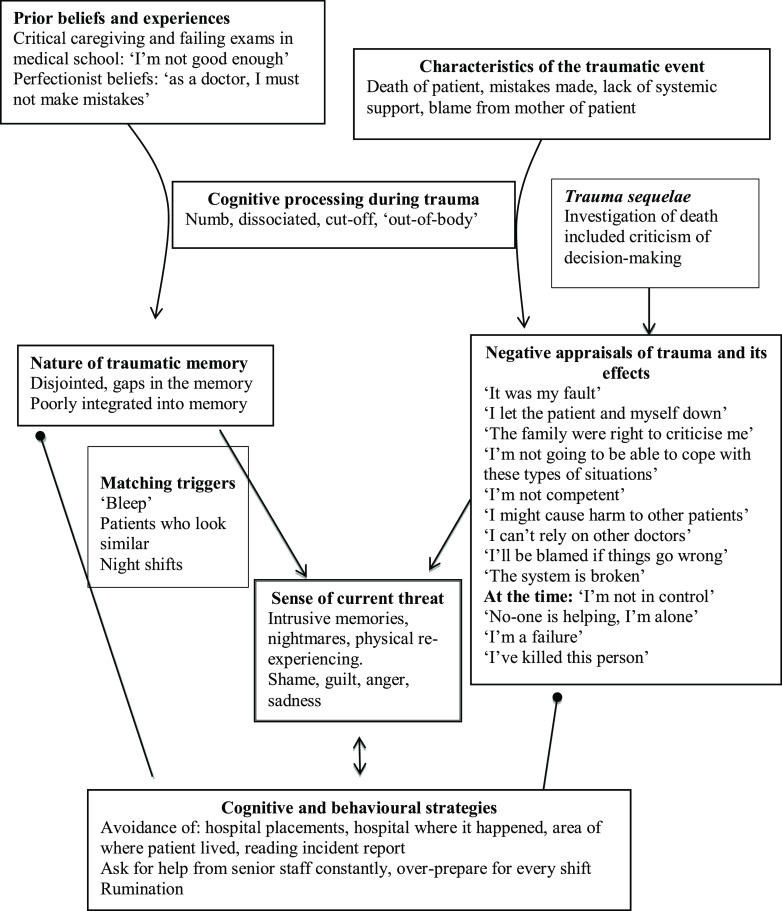
Tania’s formulation.

At the time of treatment, Tania was still working but had taken a non-clinical role to avoid the responsibility of seeing patients, and was considering a career change. She avoided the hospital where the trauma had happened and colleagues who worked there, as well as the area where the patient’s family lived, as she believed they were still angry with her. Tania had broken the news of the death to Queenie’s family, who had understandably been grief-stricken and had shouted at Tania for not having saved her. This memory, as well as images of the resuscitation, frequently recurred in nightmares and as intrusive memories during the day. Tania felt guilty, ashamed, sad and angry when she remembered the incident, and often ruminated about the mistakes she had made. Her formulation is illustrated in Fig. 2.

### Treatment

A simplified version of this formulation was developed with Tania. Psychoeducation about PTSD and moral injury was used to normalise her experiences. Tania described little interest or pleasure in activities she had previously enjoyed but was amenable to the idea of ‘reclaiming your life’ and agreed to start with going for a walk with her partner after work and watching a film at the weekend.

Tania was asked to give a brief description of the traumatic event and immediately highlighted her responsibility for Queenie’s death and her incompetence as a doctor, so this became an initial target for treatment. Tania rated herself as 100% responsible for the death, as she had missed the signs indicating that Queenie was deteriorating. Guided discovery was used to contextualise Tania’s decision-making. She had been covering two wards during a night shift and had been dealing with several very ill patients during the night. Tania had avoided reading the official report into Queenie’s death but agreed to read it together with her therapist during a session. The report was less critical than Tania had imagined and listed several factors contributing to Queenie’s death, including the failure of the day shift to spot signs of sepsis. Tania was criticised for not acting sooner when blood tests revealed a concerning marker, but the report concluded that, although mistakes had been made, the severity of the Queenie’s illness meant that her death was likely to have occurred regardless. These various factors were added to a responsibility pie chart, and Tania reported a shift in her appraisal of blame to 20%. However, she still felt this was ‘unacceptable’, and expressed the belief that doctors needed to be ‘faultless’ as their mistakes could lead to loss of life.

Tania’s perfectionistic beliefs were underpinned by childhood experiences of critical parenting, and a sense that she was not as competent as her peers, which had been forged when she failed some exams during medical school. Tania agreed to a homework task of reading a first-person account of being a junior doctor (*This is going to hurt* by Adam Kay) and spotting any ‘less than perfect’ behaviours. She also devised an anonymous survey which she distributed on a Facebook group for junior doctors, asking them about mistakes they had made, patient deaths that had occurred during their work, whether they failed any exams during medical school, and what advice they would give a fellow junior doctor who had made an error. The results were surprising to Tania; most of the responders admitted to mistakes, feelings of incompetence, and failing exams. Tania’s belief that she needed to be perfect to be a good doctor reduced from 100% to 50%.

Tania and her therapist constructed a written narrative of the traumatic event and added in hotspot updates as they went. Tania had felt numb and dissociated during the trauma, and reported several gaps in her memory. The likely series of events was constructed when a gap was found. For example, Tania was confused that she had intrusions of Queenie’s body in different positions during the resuscitation but realised this had been due to nurses moving her so that they could get better access, while Tania had left briefly to telephone the consultant, and again when she had prepared an adrenaline injection. Hotspots relating to anger at being let down by the consultant, and the failure for other members of the medical team to help were also addressed and updated. Table 2 shows a summary of Tania’s hotspot updates.

**Table 2.** Tania’s hotspots and updates

Hotspot	Thoughts	Feelings	Updates
Drawing up adrenaline	‘I’m moving too slowly’ ‘If I get this wrong, I will kill her’ ‘Why is no one else helping?’	Frustration Doubt	I did do the amount correctly – nothing bad happened due to this They should have been helping – maybe they didn’t due to lack of experience or their own anxiety I would have tried to help in their position
Mum begging to see her daughter during resuscitation	‘I should have taken her to see her child’ ‘I’m causing her more pain’	Regret Sadness Guilt	I didn’t take her in because she was hysterical and there was no nurse available to accompany her Witnessing resuscitation attempts may not always be the right thing for parents
Seeing the decorticate movements	‘She’s dead and it’s my fault’	Sadness Helpless Ashamed	She had died, and there are multiple reasons for that I wish I had acted sooner on the blood test results, but ultimately it may not have saved her; she was very ill. I am not a bad doctor. All doctors make mistakes. The reason I feel guilty and ashamed is because I care deeply about my patients; this is a sign of a good doctor. If I am incompetent, I would have failed medical school
Consultant arriving late and slowly	‘She didn’t support me’ ‘Consultants should be 20 minutes away when on call’	Angry Betrayed	She lived a long way away – this is against the rules This should be on the serious incident report Other consultants do support their juniors When I am a consultant, I won’t act this way Her being there would have made a difference to me, but it wouldn’t have saved the patient
Breaking the news to the mother	‘She’s lost her child and it’s my fault’ ‘She’s blaming me’	Sadness Guilt	It felt like she was directing this at me but I know now that she was upset at the whole team It’s understandable that she was upset. What she said at that moment may or may not be how she feels now

Tania reported a reduction in her PTSD symptoms following the memory updating process but remained troubled by a nightmare of Queenie’s mother in distress, which was accompanied by strong emotions of guilt and sadness. Tania had not seen the mother again, so her image of her was ‘frozen’ at the point of pain and anger. In therapy, Tania decided to write a letter to her (which was not sent), expressing her empathy and remorse for the loss of her daughter, and apologising for her mistake. Tania knew that the family had strong religious beliefs, so was encouraged to use an imagery exercise to visualise the mother at a later point, more at peace with the loss, being comforted by friends and family, and believing Queenie was in heaven. Tania and her therapist talked about how Tania could move forward from the incident, taking with her the responsibility that she felt, and using it to make her a better doctor and person, rather than the incident holding her back. Tania chose to write a personal blog on a website for medics about the experience of losing a patient, and about the importance of supporting each other. She also contributed to an NHS England programme to raise awareness of sepsis across the health service.

Tania continued to feel angry about the lack of support from senior staff, and the ‘blame culture’ within healthcare which had contributed to her distress following the incident. She decided to include reflections on this in the next portfolio she submitted to her medical school. She also made a personal commitment to behave differently when she was in a position of power as a consultant and to try to improve the system.

Towards the end of therapy, Tania and her therapist returned to the hospital where the trauma happened and reconstructed the events of the day. Tania had checked ahead of time who was on shift and arranged to speak to one of the nurses who had been present during the resuscitation about what had happened. Tania explained to her that she had struggled with her own responsibility. The nurse told Tania that the staff viewed her as a compassionate and conscientious doctor, and none of them held her responsible for Queenie’s death.

Tania agreed to a behavioural experiment where she took on locum shifts on a hospital ward. She practised the ‘then versus now’ stimulus discrimination technique to address triggers to her memories, including the ‘bleep’ alarm system which calls doctors to emergencies and patients who looked similar and presented with similar clinical features as the young woman who died. Her therapist helped her identify and drop safety behaviours, such as calling the consultant unnecessarily for a second opinion and over-preparing for shifts by reading textbooks and medical journals.

### Outcome

Tania reported a positive outcome to treatment. She felt ready to take on a hospital placement. Importantly, rather than being plagued with self-critical rumination, Tania was able to view herself more kindly, as someone who cared deeply about her patients and her work, qualities of a good doctor rather than an incompetent one. In a recent follow-up, Tania reported she was still doing well, had completed her medical training, and is now applying for roles as a consultant.

Tania’s scores on the outcome measures at baseline and end of treatment are presented in Table 3.

**Table 3.** Tania’s scores on standardised outcome measures

Measure	CAPS	PCL-5	BDI-II	WSAS
Baseline	34	44	23	19
End of treatment	2	0	1	2

CAPS, Clinician-Administered PTSD Scale for DSM-5 (Weathers *et al*., 2013), the maximum score is 80; PCL-5, PTSD Checklist for DSM-5 (Blevins *et al*., 2015), the maximum score is 80; BDI-II, Beck Depression Inventory – Second Edition (Beck *et al*., 1996), the maximum score is 63; WSAS, Work and Social Adjustment Scale (Mundt *et al*., 2002), the maximum score is 40.

## Key practice points

(1) Moral injury refers to psychological distress that can arise following events which have transgressed an individual’s moral and ethical code.
(2) Moral injury, where it arises with PTSD, can be formulated using Ehlers and Clark’s cognitive model of PTSD.
(3) Treatment aims to identify and address moral injury-related beliefs, correcting misappraisals and generalisation where they have occurred, and accepting responsibility or allocating blame where it is due.
(4) Other CT-PTSD techniques including updating the trauma memory, trigger discrimination, reclaiming your life and dropping unhelpful coping strategies are also used.
